# Role of the dental surgeon in the early detection of adults with underlying HIV infection / AIDS

**DOI:** 10.4317/medoral.17527

**Published:** 2011-12-06

**Authors:** Julián Campo, Jorge Cano, Jorge del Romero, Victoria Hernando, Julia del Amo, Santiago Moreno

**Affiliations:** 1 Department of Medicine and Orofacial Surgery. School of Dentistry. Complutense University. Madrid; 2 Sandoval STI Clinic. Community of Madrid; 3 National Epidemiology Center. Carlos III Health Institute; 4 Department of Infectious Diseases. Ramón y Cajal Hospital. Madrid (Spain

## Abstract

A review is made of the late diagnosis of human immunodeficiency virus (HIV) infection, a subject of growing interest in public health. It has been estimated that in Europe 30% of all HIV-infected people are unaware of their seropositive condition, and this in turn is associated with a poorer long-term disease prognosis and an increased risk of transmission to other individuals. The role of the dental surgeon in this context could be of great importance, since there are many oral lesions that can suggest the existence of underlying infection. The study also addresses the controversial subject of rapid HIV testing, and whether these tests should be performed on a routine basis in the dental clinic, or whether it is preferable to refer the patient to a specialized center.

** Key words:**HIV in Spain, HIV screening, early diagnosis.

## The late diagnosis of HIV infection in Spain; magnitude, risk factors and implications

The natural course of HIV infection has changed notoriously as a result of the introduction of combination or highly active anti-retroviral therapy (HAART), which began to be administered in Spain in 1997. Such treatment has led to a drastic reduction in mortality associated to the acquired immunodeficiency syndrome (AIDS), and to increased survival among HIV-infected individuals HIV (1). In the year 2009, the HAART response rate was very high, affording sufficient host immune recovery to allow the control of opportunistic infections, thanks to the introduction of increasingly well tolerated and simpler treatments such as the combination of several drug substances in a single tablet (2).

However, despite initiatives to promote the early diagnosis of HIV infection in Europe in the last decade, studies suggest that there is still a significant proportion of individuals who are not aware of their seropositive condition (3). In Europe, it has been estimated that 30% of all HIV-positive subjects are unaware that they have been infected (4), and that these individuals could be responsible for almost 60% of all new infections (5). At present, most cases of HIV infection in Spain are found in heterosexuals and male homosexuals, and to a lesser degree in intravenous drug abusers. Furthermore, at least 30% of all new diagnoses corre-spond to individuals born outside the country (2,6). In relation to the late diagnosis of HIV infection in Spain (CD4+ lymphocyte count < 350 cells/mm3), most cases correspond to males over 50 years of age, intravenous drug abusers or heterosexuals, and people born outside the country (6). Based on this definition, and analyzing the data of the adult seropositive cohort of the AIDS Investigation Network (Red de Investigación en SIDA, CoRIS), the late diagnosis rate is about 48%, and affects mainly males over 50 years of age, heterosexuals, and people born outside of Spain (7).

These observations have important clinical and public health implications. Late diagnosis is clearly associated to a greater risk of progression towards AIDS and to increased morbidity and mortality. In addition, HAART toxicity increases in the presence of lower CD4+ lymphocyte counts at the start of therapy. Lastly, and no less importantly, a delayed diagnosis implies a greater risk of inadvertent HIV transmission (5,8).

Thus, a delay in diagnosing the disease is one of the main challenges of the HIV epidemic today. The definition of “late diagno-sis” has been the subject of debate and controversy, though in Europe the term is taken to imply patients who at the time of diag-nosis present CD4+ lymphocyte counts of < 350 cells/mm3, or show clinical manifestations of AIDS (9).

## Strategies for reducing the late diagnosis of HIV infection: the role of the dental surgeon

Traditionally, HIV testing has been offered to people and population groups at risk of becoming infected with the disease, such as intravenous drug abusers, male homosexuals or immigrants from countries in which the infection is very prevalent. Some studies indicate that only one-third of all patients seen in the healthcare services with suggestive clinical processes (clinical indicators) or with risk behavior or risk factors are subjected to serological testing for HIV (10). In addition, between 10-25% of all seropositive individuals do not refer antecedents of risk behavior (11).

A number of initiatives have been launched in recent years to improve the diagnosis of HIV infection. In the year 2006, the United States Centers for Disease Control (CDC) published recommendations for increasing the early diagnosis of the disease and for expanding the role of the healthcare professionals in reducing the high rates of late diagnosis (12). These guidelines suggest that healthcare professionals should recommend HIV testing in all people between 13-64 years of age, as part of routine healthcare practice. In order to eliminate potential barriers against HIV testing, the CDC also proposed obviating the need for a “counseling pretest” and signed informed consent form specific for HIV testing – without this meaning that the test is no longer voluntary.

Posteriorly, the WHO/UNAIDS (13), some national health agencies (14), and many medical societies (15), unanimously recommended the routine prescription of HIV tests in patients with clinical markers suggestive of infection (i.e., with disorders “indicative” of underlying HIV infection), or with individual behavioral or other risk factors.

Recently, the European Center for Disease Prevention and Control (ECDC), through its website (www.ecdc.Europe.eu), has published a series of guidelines on HIV testing in Europe (16).

The strategy adopted by the CDC is to perform generalized HIV testing of all people accessing the healthcare system for any reason, and a number of studies have established that this measure would be cost-effective in regions where the prevalence of undiagnosed HIV infection is over 0.1% (17). Cost-efficacy studies are being carried out in the United Kingdom, and this strategy has also been adopted in France with the purpose of reducing delays in diagnosis (14,18). In Spain a study has been made in Primary Care Centers of the Community of Madrid, in the context of an infectious diseases serovigilance survey – the observed prevalence of hidden (i.e., undiagnosed) HIV infection being about 0.35% (95%CI 0.13-0.57%)(19).

In any case, it is important to distinguish between HIV testing offered to people with known risk behaviors or as part of an ante-natal screening program in pregnant women and a diagnosis derived from the actual presence of clinical signs or symptoms of underlying HIV infection. It is in this latter scenario where dental surgeons as well as other healthcare professionals may play an important role. Many clinical and behavioral indicators of underlying HIV infection ( Table 1 and  Table 2) can be used by dental pro-fessionals with the purpose of referring a patient for HIV testing or of performing rapid HIV testing in the dental clinic itself (2).

**Table 1:**
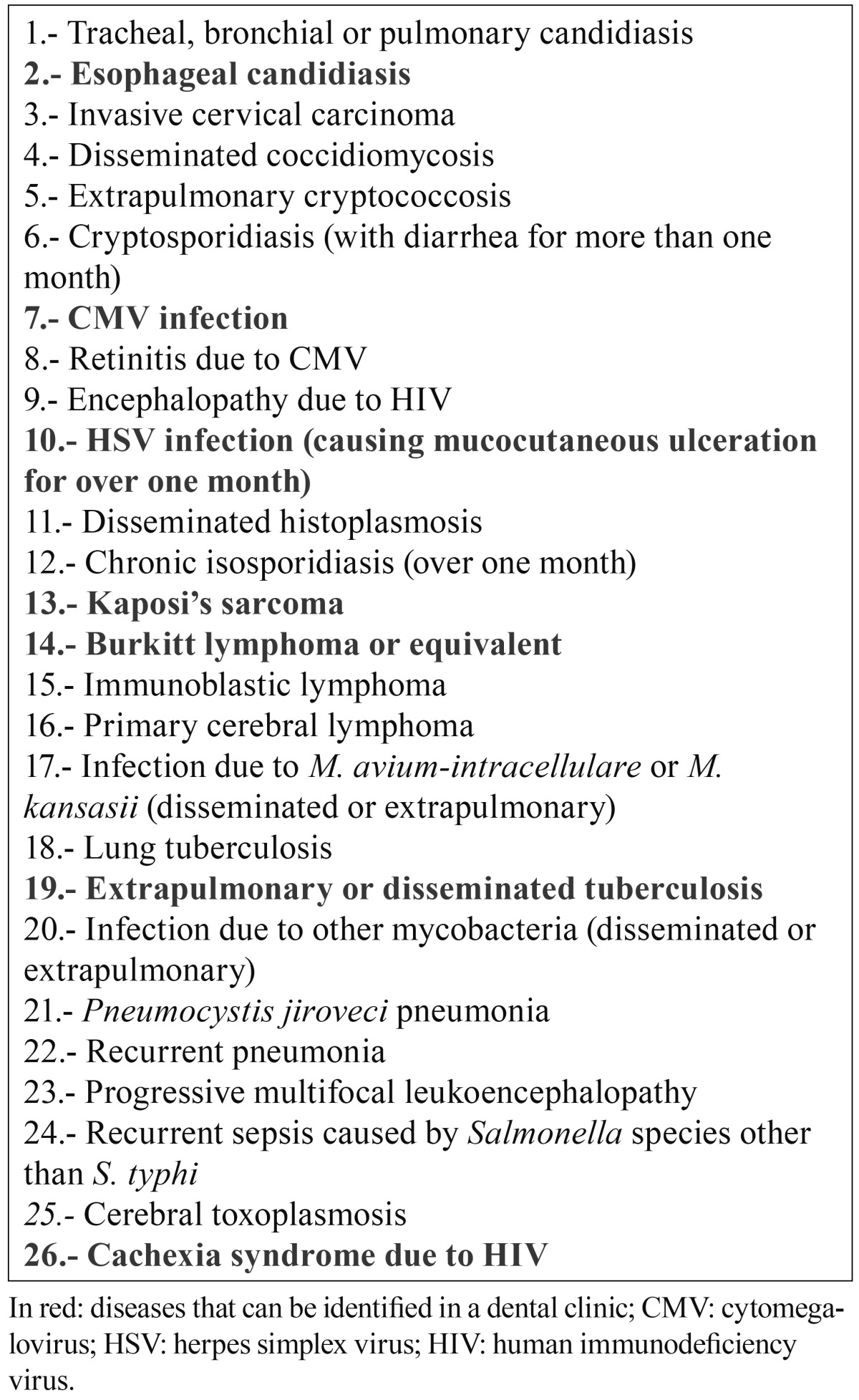
Opportunistic infections or neoplasms suggestive of AIDS in which HIV testing ALWAYS must be recommended (2).

**Table 2:**
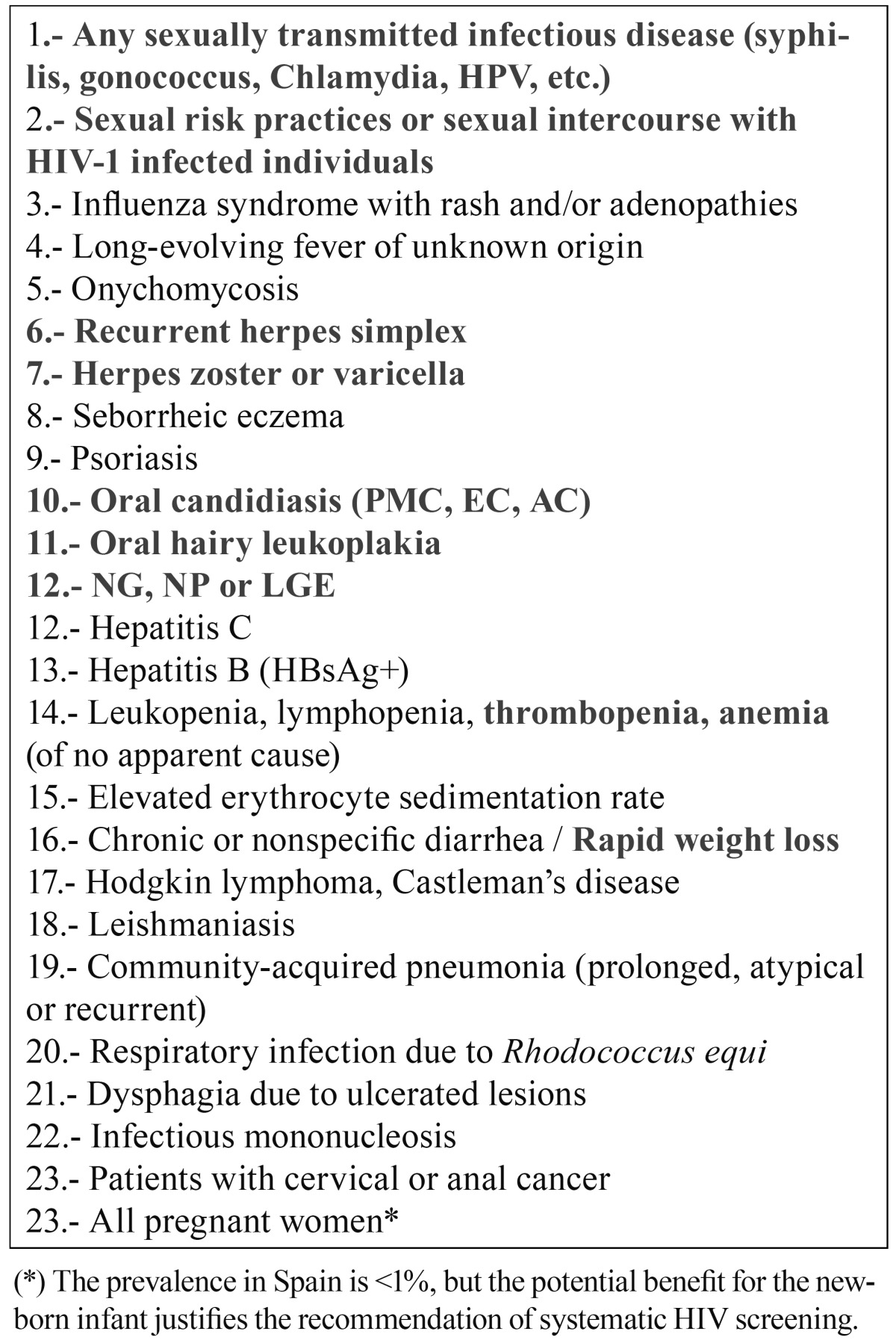
Situations in which HIV screening is recommended (2).

Specific platforms have been created both in Europe (“HIV in Europe”) and in Spain (“HIV in Spain 2009 and 2010”) to promote the early diagnosis of HIV, with the support of the health authorities and the political and social leaders. Specifically, in Spain these programs for the early detection of HIV infection are supported by the Secretariat of the National AIDS Plan (2), and are targeted to all potentially implicated healthcare professionals – including dental surgeons.

Among the main conclusions drawn by the members of the “HIV in Europe” platform held in 2007, mention should be made of the following recommendations for HIV screening in Europe:

(3)All healthcare professionals should be aware of the need to perform HIV tests in more individuals, and should be familiarized with the range of lesions in which the prevalence of underlying HIV infection justifies patient referral for testing.

(4)Certain healthcare professionals such as general practitioners, dentists, dermatologists, specialists in sexually transmitted diseases, gynecologists and emergency care physicians should be specific targets for this type of initiative, since they are more likely to be the first to encounter HIV-infected patients with comorbidities (8).

Furthermore, in the context of dental practice, the committee of the “HIV in Europe” platform considers that as long as data are pending on the prevalence of HIV infection among patients with such comorbidities, the presence of oral manifestations suggestive of underlying HIV infection is indicative of the need to perform diagnostic tests (8).

In addition, it must be taken into account that adults over 20 years of age tend to visit their dentist more often than their physician (20); as a result, dental surgeons may have an important opportunity to detect underlying HIV infection (21).

## Orofacial clinical indicators suggestive of HIV infection

From the start of the HIV epidemic, the oral cavity has played an important role in monitoring the progression of HIV infection through the appearance of specific lesions – fundamentally oral candidiasis (OC) and oral hairy leukoplakia (OHL), which have been closely correlated to low CD4+ lymphocyte counts and high plasma viral loads (22-24). Thus, it has been estimated that over 90% of all AIDS patients present one or more oral manifestations during the course of the disease, and these manifestations are moreover often the first sign of immune depression (25).

On the other hand, the prevalence of specific lesions such as OC, OHL and Kaposi’s sarcoma (KS) is diminished in patients subjected to highly active antiretroviral therapy (HAART)(26,27), while other oral lesions such as papillomas and salivary gland disorders are increased in such individuals - thus suggesting that they may form part of an immune reconstitution syndrome (IRS), secondary to the start of HAART (22,28).

In London in 1992 the World Health Organization (WHO) and the EC -Clearinghouse developed a classification based on the prevalence of oral manifestations in HIV-positive patients. Three groups were established, and in Group 1, corresponding to oral lesions commonly associated with HIV infection, the following conditions are enumerated: pseudomembranous candidiasis (PMC); erythematous candidiasis (EC); angle cheilitis (AC); oral hairy leukoplakia (OHL); necrotizing gingivitis (NG); necrotizing periodontitis (NP); linear gingival erythema (LGE); Kaposi’s sarcoma (KS); and non-Hodgkin lymphoma (NHL)(29).

Recently, the Oral HIV/AIDS Research Alliance (OHARA), which forms part of the AIDS Clinical Trials Group (ACTG) and was created in 2006 to investigate all orofacial aspects of HIV infection, has published an update on the definitions of the oral manifestations of HIV-positive patients. The update is based on the 1992 classification of the EC – Clearinghouse, and contem-plates the clinical descriptors of the Group 1 oral lesions (1992) plus other conditions such as oral papilloma, labial herpes, recurrent intraoral herpes and recurrent aphthous stomatitis (since these disorders are very prevalent in such patients). In addition, it addresses the symptoms of the patients and the duration of the disease, if known. On the other hand, lesions such as “nonspecific ulcerations” (NOS) have been merged with “necrotizing ulcerative stomatitis” in order to facilitate the diagnosis for non-expert professionals. In the same way, NG and NP have been merged, since they cannot be distinguished by visual inspection if X-rays and periodontal probing are not available. Another proposed change is addition of the definition of oral squamous cell carcinoma (OSCC) associated to HIV, since there is evidence of an increased risk of OSCC in HIV-positive individuals versus the general population. On the other hand, division of the section “Salivary gland disease” into two separate categories - “salivary hypofunction” and “salivary gland swelling” - has been proposed, since they may manifest independently in one same patient (22).

A number of studies have reported that Group 1 oral lesions, alone or in combination, could be used as diagnostic markers in the screening of patients suspected to be seropositive for HIV (30,31), particularly in centers with limited resources, or where patients are reluctant to undergo HIV testing (32).

In 1998, Robinson et al. (32) evaluated the usefulness of oral lesions as predictors of underlying HIV infection in populations with different prevalences of the infection. In the general population, where the prevalence of HIV infection is low, the clinical diagnosis of Group 1 oral lesions alone was seen to be a poor predictor of HIV infection, with many false positive results. However, the positive predictive value (PPV) increased on using information from the medical records, such as for example infection risk behavior. Thus, only 2.6% of the individuals in England and Wales presenting oral lesions similar to OHL would have HIV infection, but in the case of a homosexual male the PPV would increase to 57.4%, and to 73.8% in the city of London (32).

In 2008, Bhayat et al. (30) carried out another study attempting to predict HIV infection among dental patients who were unaware of their seropositivity. They found that if a patient presented NG, the probability of HIV infection was 40 times greater than in the absence of NG (30).

This same group recently carried out a study to determine the prevalence of HIV infection and of Group 1 oral lesions among adults seen in a Primary Care Center in South Africa. They examined a total of 522 patients, and the prevalence of HIV infection was found to be 40%. Of these patients, 53% presented some Group 1 oral lesion – PMC and EC being the most common findings. The Odds Ratio (OR) of having HIV infection in the presence of OHL was 38, versus 78 in the case of multiple PMC plus OHL lesions. In patients with combinations of multiple oral lesions, the mean positive predictive value (PPV) and negative predictive value (NPV) was 91.7% and 61.2%, respectively. The authors therefore concluded that Group 1 oral lesions alone or in multiple combinations can be reliably used as HIV screening tools, particularly in centers with limited resources, or where testing is little used or is very expensive (33). In addition, they pointed out that the usefulness of these Group 1 oral lesions is limited by clinician skill in being able to recognize and correctly diagnose them. Thus, correct clinician training in the diagnosis of these lesions is very important, since it may increase the number of patients referred for HIV screening and avoid a late diagnosis of this infection.

**Figure 1:**
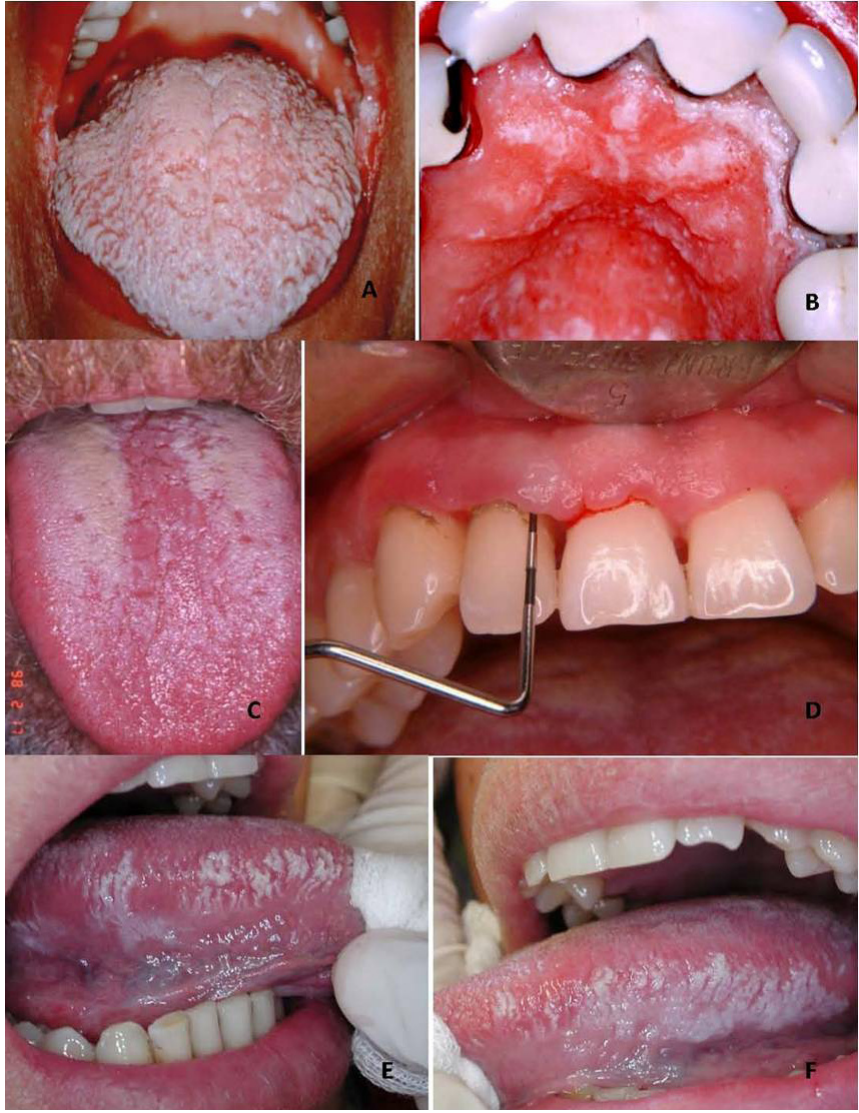
Oral lesions found to be highly prevalent in HIV-positive patients. A) Pseudomembranous candidiasis (PMC) of the tongue and bilateral angle cheilitis. B) PMC of the palate in the same patient as before (multifocal candidiasis). C) Erythematous candidiasis (EC). D) Necrotizing gingivitis. E) Oral hairy leukoplakia (right lateral margin of the tongue). F) Oral hairy leukoplakia in the same patient (left lateral margin).

**Figure 2:**
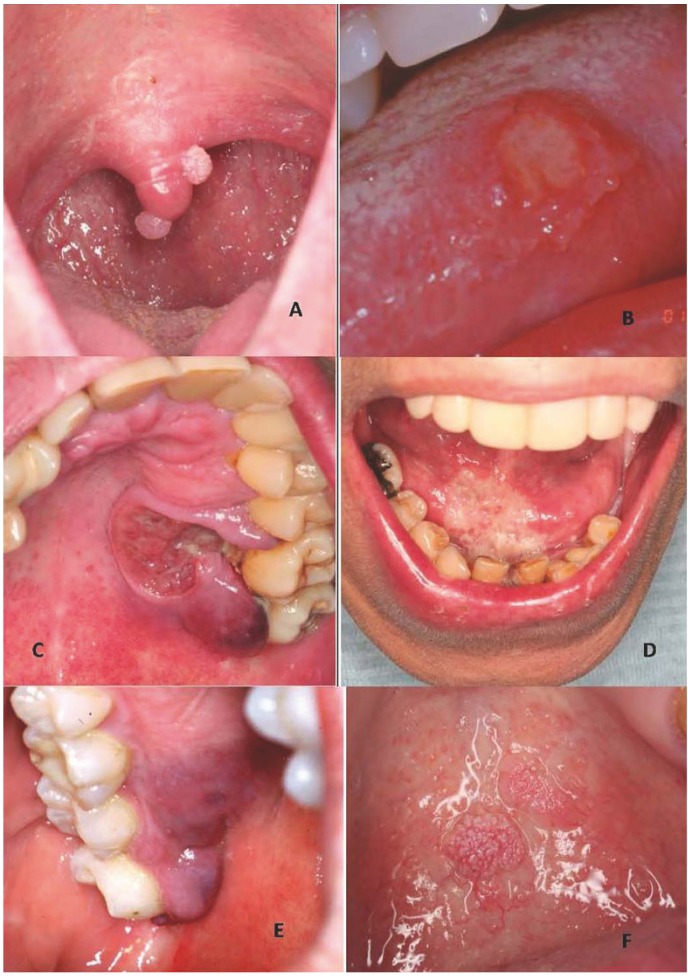
Other oral lesions that may be found in HIV-positive patients. A) Human papillomavirus (HPV) lesion. B) Lesion of the lateral margin of the tongue due to recurrent aphthous stomatitis. C) Plasmablastic lymphoma of the palate. D) Secondary oral tuberculosis lesion in an undiagnosed HIV-positive patient. E) Kaposi’s sarcoma of the gums. F) Oral condyloma lesion of the palate.

Based on the above data and on the review of the literature,  Table 3 proposes a series of orofacial manifestations suggestive of underlying HIV infection, including not only the commented Group 1 oral lesions but also the suggestions of the OHARA and those oral manifestations of the most common sexually transmitted infections which we feel to justify the decision of the dental surgeon to refer the patient for HIV testing ( Table 3)(Figs. 1,2).

**Table 3:**
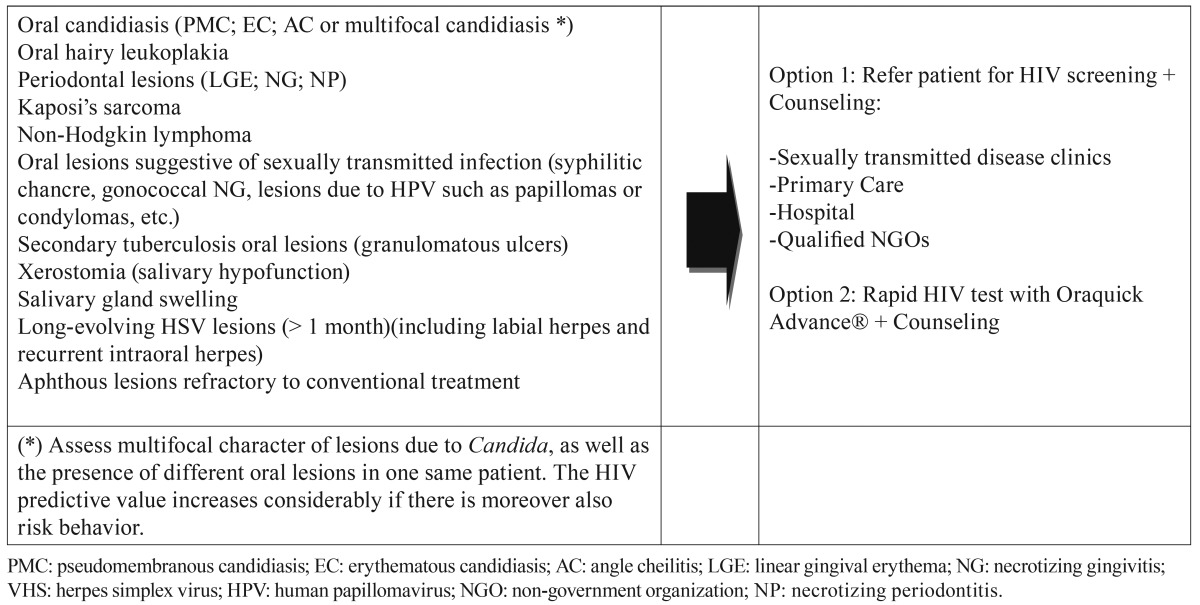
HIV screening proposal based on orofacial manifestations suggestive of underlying HIV infection.

## Rapid HIV testing in dental practice. Controversial aspects

The CDC recommends that in order to improve prevention and the results of combination antiretroviral therapy (HAART), and to reduce HIV transmission, the places where testing can be made should be diversified beyond the usual settings – this in some instances including the use of rapid tests (34). 

Routine HIV diagnostic tests are predominantly carried out using blood samples, and the results are obtained within 10-14 days. Almost 30% of all tested individuals fail to report to pick up their test results (35).

A rapid HIV detection test known as the Oraquick Advance® (Orasure Technologies, Bethelem, PA, USA) has been approved by the United States FDA, and can yield results in 20 minutes. This test detects antibodies against HIV-1 and HIV-2 in gingival crevicular fluid (GCF)(transudate), in whole blood from a finger prick sample, in whole blood drawn from a vein, and in plasma samples, based on ELISA technology (21). The test can be used outside the traditional clinical office setting in the United States, provided the clinician holds a special certificate from the CLIA (Clinical Laboratory Improvement Amendments) and is trained in performing the test and in interpreting the results (35). As a result, this test could easily be used in a dental clinic using GCF samples. In Spain a number of non-government organizations (NGOs) have been using the test for several years.

The mentioned rapid test was evaluated by Debattista et al. (2007) and by Wesolowski et al. (2006), yielding a mean specificity and sensitivity of 100% and 98.3%, respectively, and having been used in screening studies in a number of countries (36,37). The reviewed studies that have used this diagnostic kit have reported specificity values of between 99-99.87%, and sensitivity values in the range of 96-100% (38,39).

False positive readings are rare, particularly when sampling has been correctly performed. In this context, the sample consists of crevicular fluid (not saliva), which is produced within the gingival sulcus between the tooth and the gums. It has been estimated that there may be 1-2 false positive readings per 1000 tests. If incorrect sampling is discarded, such false positive results may be associated to the presence of antibodies in the GCF targeted to other viral infections such as Epstein-Barr virus or hepatitis A or B. Negative results should be regarded as definitive (35). It is important to emphasize that a point in favor of such testing is the fact that the dental surgeon precisely knows the origin and the composition of the crevicular fluid sample. This greatly facilitates correct learning of the technique, eliminating errors that could occur with other professionals by confusing crevicular fluid with normal saliva – the origin and composition of which is different (21). However, in addition to this aspect, it must be taken into account that any HIV test must be accompanied by patient information and consent both before and after the test.

It is here where the discrepancies and controversial issues appear. In effect, in a country such as Spain, where almost all dental care is private, the following questions could be raised, as has already been done by other authors in the United States (21):

- Would dental surgeons have the ethical and professional responsibility to offer rapid HIV tests to all patients, considering the public health problem posed by the fact that many HIV-infected individuals are unaware of their seropositivity?

- Do dental surgeons have the training needed to perform the rapid diagnostic test and interpret its results?

- Do dental surgeons have to offer patients pretest counseling and/or information?

- Should they refer the patient to the public healthcare system for HIV testing in the presence of the abovementioned clinical and behavioral indicators?

In relation to this subject, mention should be made of the many barriers facing rapid HIV testing in the dental clinic, and which could be extrapolated to the situation found in our country (40): (i) shortcomings in terms of knowledge and adequate training in rapid HIV testing among dental professionals; (ii) fear, concern or inexperience in giving bad news to patients; (iii) a lack of interest in such testing among dental professionals; (iv) the perception that HIV testing is not a competence of the dental surgeon; (v) reluctance on the part of the patient to undergo such a test in a dental clinic; and (vi) economical and time issues.

On the other hand, it must be taken into account that dental professionals in Spain work at different levels – fundamentally in private practice, but also in Primary Care (PC), University academic centers, and increasingly also in already established Hospital Departments of Stomatology and existing and/or future Hospital Odontology Units.

This situation raises the hypothesis that rapid HIV testing would be best performed by dental professionals within the public healthcare system, particularly dentists in Primary Care, after receiving adequate specific training in the technique and in the pretest information given to patients. Testing in this scenario would be justified by the fact that many patients visit a dentist more often than a physician. Another aspect to be considered is whether it would be more advisable to include these aspects and issues in the university training of future dental surgeons – most of which will ultimately work in private practice. In this context, the data obtained by Patton et al. in 2002 in American universities are not very encouraging, since only 33% of the Dental Schools were found to include the legal aspects of HIV screening in their curricula, and only 15% informed their students of when to refer a patient for HIV testing. Moreover, 63% of the surveyed Dental Schools did not offer HIV testing to their own patients (40).

Considering the above and based on the existing information, we consider that the dental surgeon, in the same way as in screening for oral cancer, could be a key element in the early diagnosis of patients with underlying HIV infection. Since the oral cavity is very accessible, and many patients visit their dentist more often than their personal physician, we feel that detection of the oral indicators proposed in  Table 3 would be strongly suggestive of the need to refer the patient to Primary Care, to a center specialized in sexually transmitted diseases, or to a hospital center for due confirmation of HIV infection and patient counseling.

In the not too distant future, dental professionals in our country might adopt a more active role in the early detection of HIV infection – though this will require all aspects related to the rapid test to become incorporated as part of the global competences of the dentist, in the same way as performing a biopsy when oral cancer is suspected. In this sense the study plans of our Dental Schools will have to be updated, incorporating this new function to further implicate dental surgeons in public health matters. Until then, a good option would be to inform and enhance awareness among the dental professionals of the problem posed by late or delayed diagnosis in HIV infection and its healthcare consequences, through the Professional Dental Colleges network (2), the General Council, Dental Schools, Scientific Societies, journals and webpages of our discipline. Such information should be conveyed not only to dental professionals but also to patients who thus may begin to regard their “usual dentist” as a healthcare professional concerned about their general health – not only their dental health.
